# Recommendations for clear aligner therapy using digital or plaster study casts

**DOI:** 10.1186/s40510-018-0224-2

**Published:** 2018-07-19

**Authors:** Hsiu-Ching Ko, Weitao Liu, Derek Hou, Sepideh Torkan, Charles Spiekerman, Greg J. Huang

**Affiliations:** 1 Department of Orthodontics, School of Dentistry, University of Washington, 1959 NE Pacific ST, Health Sciences Center, D-569, Box 357446, Seattle, WA 98195-7446 USA; 2 Department of Biostatistics, School of Public Health, University of Washington, Seattle, USA

## Abstract

**Background:**

Clear aligner therapy has evolved considerably since its introduction 20 years ago. Clinicians have become more experienced with aligner therapy, but little is known about the types of malocclusions that clinicians currently treat with aligners. Similarly, it is not known if viewing digital vs plaster models has any impact on the treatment planning process for aligners. The aim of this study was to assess which types of malocclusions are recommended for treatment with clear aligners, and also to determine if recommendations for aligner treatment differed when using digital versus plaster models.

**Methods:**

Sixteen orthodontists treatment planned 20 cases at two time points with either the same or different model formats (digital versus plaster). As part of the treatment planning process, they were asked whether each patient was a good candidate for Invisalign® treatment, and if not, why. Generalized estimating equations regression (GEE), the permutation test, and a logistic regression model with GEE were used to analyze the data.

**Results:**

No significant difference was found between the Invisalign® choices in the digital model group and those in the plaster model group at T1 (*p* = 0.59). There was no significant difference between the agreement rate of the different formats group and that of the same format group (*p* = 0.97). Cases with extractions had less Invisalign® recommendations (15%) compared to cases with no extractions (55%) (*p* = 0.0015). Cases with surgery had less Invisalign® recommendations (29%) compared to cases with no surgery (57%) (*p* = 0.035).

**Conclusions:**

In this study, viewing orthodontic records with digital versus plaster models did not influence decisions about Invisalign® recommendations. Additionally, the orthodontists in this study tended to not recommend Invisalign® for extraction cases, surgical cases, or difficult cases.

## Background

Orthodontic patients increasingly expect more esthetic and comfortable treatment options. Clear aligners meet these criteria, but many orthodontists do not feel that they can be utilized for all patients [1, 2]. Studies have shown that aligners are able to perform some types of orthodontic tooth movement successfully, while more complex movements, like extrusions, anteroposterior correction, and severe rotations, pose more challenges [3–5]. During the past few years, Align Technology has attempted to address these concerns by introducing improvements to the Invisalign® product that allow for better control tooth movement. The effect of these improvements on clinician’s attitudes towards cases that can be successfully treated with aligners has not yet been investigated.

Along with the increasing prevalence of aligner therapy, digital models are gradually replacing plaster models and will eventually become the new standard for orthodontic records. However, many orthodontists still find familiarity in plaster models that can be physically articulated and measured, and studies have reported that models are the single most important orthodontic record for treatment planning [6, 7]. Several studies have looked at the accuracy of digital models, and in general, they have been found to be reasonably accurate compared to plaster models [8–12]. However, more importantly, it is not known whether assessing a case with digital instead of plaster models may influence the final treatment plan.

The purpose of this study was to assess which types of malocclusions were or were not recommended for treatment with clear aligners and also to determine if these decisions differed when using digital versus plaster models.

## Methods

This study was approved by the Human Subjects Division at the University of Washington. We recruited 16 orthodontists, all of whom were in practice for a minimum of 5 years and did not routinely use digital models (other than for Invisalign® cases) (Table 1). The orthodontists were shown how to navigate the OrthoCad software (Align Technology, San Jose, CA) prior to viewing the records in the study. The orthodontists assessed a total of 20 cases at the beginning of the study (T1) and reassessed these same 20 cases again after at least 3 months had passed (T2).

**Table 1:** The demographic characteristics of the orthodontists

Group of practitioners	1	2
	Same format of models	Different formats of models
Gender		
Male	6	4
Female	2	4
Years in practice		
Average	22.8	27.6
< 10	2	1
11~20	1	1
21~30	2	3
> 30	3	3

**Fig. 1:**
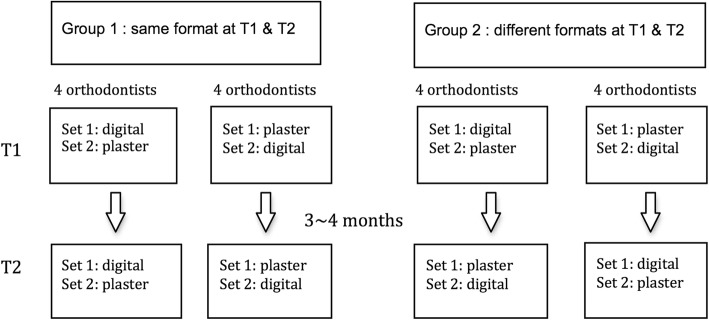
Scheme for assignment of orthodontists and cases

Orthodontists were divided into two groups based upon matching for gender and years in practice (as well as possible). The demographics of the two groups are shown in Table 1. One group was shown the same format models at the two time points, and the second group was shown different format models at the two time points. The pre-determined assignment scheme (shown in Fig. 1) ensured that all combinations of treatment planning with either digital or plaster models were equally distributed over the two time points. This information was used to determine if the type of model used affected the decision regarding the use of Invisalign®.

Ten different case types were planned to be included in the study to represent the breadth of cases typically seen in practice, such as Class I with various degrees of crowding, Class II division 1, Class II division 2, Class III, and open bite. We purposely weighted the sample towards cases with at least moderate difficulty, as they would offer better ability to discriminate between aligner recommendations. A list of all patients started in 2012 at the UW Graduate Orthodontic Clinic patient population was reviewed to identify cases that matched the categories planned to be included in the study. Ultimately two sets of ten cases each were selected based on matching of age, gender, A-P, vertical, transverse, and amount of crowding. A summary of these cases is presented in Table 2.

**Table 2:** Summary of patient characteristics

	Pair	Set 1			Set 2			Main condition of interest
		Case no.	Age	Gender	Case no.	Age	Gender	
Class I	1	1-1	15 years and 1 month	F	1-2	16 years and 6 months	F	Crowding
	2	2-1	14 years and 5 months	M	2-2	14 years and 9 months	M	Crowding
	3	3-1	21 years and 9 months	M	3-2*	22 years and 11 months	M	Adult; posterior crossbite
	4	4-41	13 years and 7 months	M	4-2	14 years and 2 months	M	Anterior open bite
Class II div 1	5	5-1	13 years and 4 months	F	5-2	13 years and 5 months	F	Crowding
	6	6-1	13 years and 9 months	F	6-2	12 years and 8 months	F	Deepbite
Class II div 2	7	7-1	14 years and 1 month	F	7-2	12 years and 7 months	F	Adolescent; deepbite
	8	8-1	30 years and 9 months	F	8-2	28 years and 9 months	F	Adult
Class III	9	9-1	15 years and 5 months	F	9-2	15 years and 1 month	F	Mild A-P discrepancy
	10	10-1	16 years and 6 months	F	10-2	16 years and 7 months	F	Severe A-P discrepancy

*This patient had a Class II posterior malocclusion

**Fig. 2:**
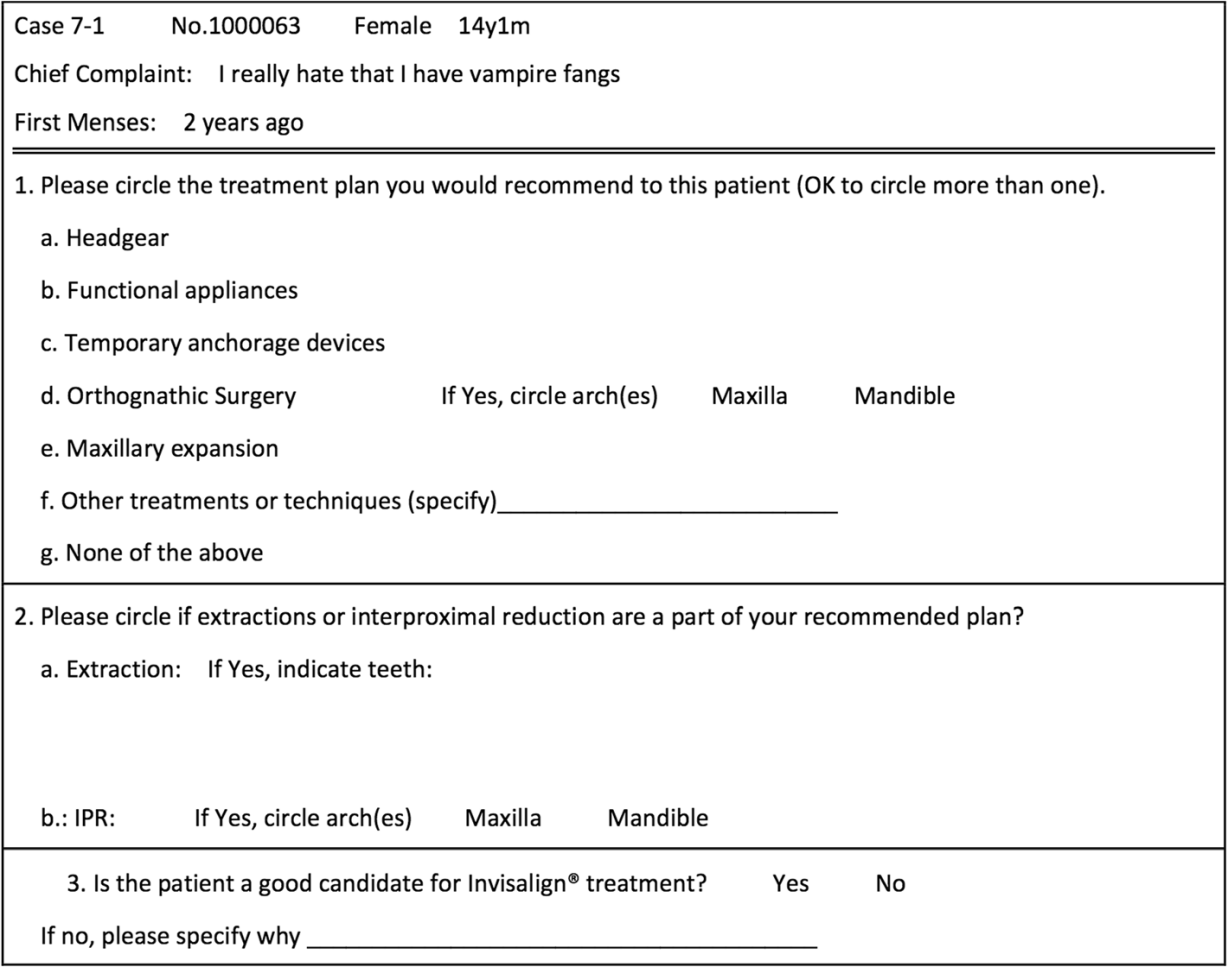
Example data collection form

The records of these 20 cases were assembled and imported into Dolphin Imaging (Patterson Dental, Chatsworth, CA) to be accessed during the study, and included intra- and extra-oral photographs, as well as the panoramic and lateral cephalometric radiographs. The plaster models were scanned using the iTero HD 2.9 intra-oral scanner (Align Technology, San Jose, CA), and the digital casts were shown using OrthoCad software (Align Technology, San Jose, CA). All patient identifiers were removed, and only study numbers were used during the evaluation by orthodontists. Subject’s eyes were blocked out in all facial photographs. A standardized form was used for treatment planning each patient (Fig. 2).

At the initial treatment planning session, orthodontists were shown the intra- and extra-oral photos, panoramic radiographs, lateral cephalometric radiographs, and the study models in either digital or plaster format. After at least 3 months had passed the orthodontists were asked to treatment plan the same cases again, using either digital or plaster models based on the pre-assigned scheme (Fig. 1).

### Statistical analysis

A generalized estimating equations regression was utilized to determine if there was a difference in the recommendation for Invisalign® treatment based upon the type of model (plaster versus digital) that was viewed. The permutation test was utilized to assess if there was a difference between the agreement rate of those who viewed the models twice with different formats and that of those who viewed the models twice with the same format. The level of significance was set at *p* < 0.05. Additionally, a logistic regression model with GEE was utilized to help explain why Invisalign® was not recommended as a treatment option.

## Results

There was no significant difference between the percentage recommending Invisalign® in the digital model group and that in the plaster model group at T1 (*p* = 0.59). In the digital model group, 75.6% of initial treatment plans at T1 did not recommend using Invisalign®, while this rate was 76.9% in the plaster group (Table 3).

**Table 3:** Percentage of decisions aligners not recommended at T1

Invisalign® recommendation	Models		Difference	95% confidence interval of difference		*p* value
	Digital	Plaster				
% of decisions not recommending	75.60%	76.90%	1.25%	− 3.30%	5.80%	0.59

Additionally, there was no significant difference between the agreement rate of those who viewed models twice with different formats and that of those who viewed models twice with the same format (*p* = 0.97). Thus, the percentages of cases that practitioners felt could be treated by Invisalign® did not appear to be influenced by the manner (plaster versus digital) in which the models were presented (Table 4). Therefore, we decided to pool the recommendations from all 16 assessors at the first assessment time point in order to assess reasons for not recommending aligners.

**Table 4:** Intra-rater agreement when viewing the case twice

Invisalign® recommendation	Models		Difference in agreement	95% confidence interval of difference		*p* value
	Different formats	Same format				
Same decision at T1 and T2	82%	81%	1%	− 21%	20%	0.97

Table 5 reports the case characteristics, number and percentage of surgical plans, extraction plans, and Invisalign® recommendations. The main reasons are also listed for each case. (The maximum number of recommendations for each parameter is 16.)

**Table 5:** Characteristics, treatment plans, and Invisalign® recommendations

Category	Case no.	Main characteristics	Times surgery recommended (%)	Times extraction recommended (%)	Times aligners not recommended (%)	Main reasons for not recommending aligners (times mentioned)
Class I	1-1	Moderate crowding, protrusive profile	0	15 (94%)	14 (88%)	Extraction case (10)
	1-2	Moderate crowding, Mx right lateral crossbite Straight profile	0	3 (19%)	6 (38%)	Extraction case (2) Crossbite (2)
	2-1	Moderate to severe crowding; High canine	0	12 (75%)	13 (81%)	Extraction case (7) Rotation (3)
	2-2	Moderate to severe crowding, Mx laterals in crossbite	0	14 (88%)	14 (88%)	Extraction case (9) Difficult case (2)
	3-1	Adult Severe crowding Bilateral posterior crossbite	2 (12%)	12 (75%)	15 (94%)	Extraction case (6) Difficult case (3) Surgery case (2) Crossbite (2)
	3-2*	Adult Unilateral posterior crossbite, severely proclined upper incisors, class II	8 (50%)	13 (81%)	15 (94%)	Extraction case (6) Surgery case (5) Difficult case (2)
	4-1	Anterior open bite, facial asymmetry	7 (44%)	9 (56%)	14 (88%)	Surgery case (6) Extraction case (3) Anchorage (2) Open bite (2)
	4-2	Anterior open bite Mild crowding, protrusive lips	1 (6%)	15 (94%)	14 (88%)	Extraction case (7) Surgery case (2) Difficult case (2) Open bite (2)
Class II Div 1	5-1	Moderate crowding	0	13 (81%)	14 (88%)	Extraction case (11)
	5-2	Moderate crowding	1 (6%)	15 (94%)	14 (88%)	Extraction case (6) Difficult case (2) Surgery case (2) Anchorage (2)
	6-1	Deepbite Full-cusp class II Retrognathic mandible	8 (50%)	11 (69%)	13 (81%)	Surgery case (5) Extraction case (4) Difficult case (2) Anchorage (2)
	6–2	Deepbite Half-cusp class II;	1 (6%)	2 (12%)	9 (56%)	Deepbite and deep COS (3) Extraction case (2) (9 suggested functional appliances)
Class II Div 2	7-1	Deepbite, maxillary moderate crowding	1 (6%)	4 (25%)	7 (44%)	Extraction case (2) (3 suggested functional appliances)
	7-2	Deepbite	2 (12%)	0	12 (75%)	AP correction (5) (10 suggested functional appliances)
	8-1	Adult Deepbite	8 (50%)	3 (19%)	9 (56%)	Surgery case (3) Extraction case (3)
	8-2	Adult Severe crowding	3 (19%)	13 (81%)	14 (88%)	Extraction case (7) Surgery case (3) Difficult case (2) Anchorage (2)
Class III	9-1	Mild A-P discrepancy Asymmetry	12 (75%)	1 (6%)	9 (56%)	Surgery case (6) AP correction (2)
	9-2	Mild A-P discrepancy Asymmetry	2 (12%)	0	8 (50%)	Severity (3) AP correction (3)
	10-1	Anterior crossbite, severe A-P discrepancy Asymmetry	10 (63%)	4 (25%)	15 (94%)	Surgery case (7) Difficult case (4) Anchorage (2)
	10-2	Anterior crossbite, severe A-P discrepancy Asymmetry	13 (81%)	3 (19%)	15 (94%)	Surgery case (10) Deepbite or deep COS (2)

*This patient had a Class II posterior malocclusion

In the Class I category, it was evident that cases requiring extractions were not judged to be good candidates for Invisalign®. Two Class I open bite cases (recommended extractions by 56% and 91% of the judges, respectively) also were not judged to be good candidates for Invisalign®. Only one of the subjects in the Class I category was judged to be a reasonable candidate for Invisalign® (case 1-2), and the percentage of the raters who recommended extraction for this patient was the lowest in this category (19%).

In the Class II division 1 category, extractions were included in the treatment plans of the majority for patients other than patient 6-2. This subject had minimal crowding and only a half-cusp A-P discrepancy. Almost half of the practitioners felt this case could be treated with Invisalign®.

The two patients in the adolescent Class II division 2 category did not have high extraction rates. However, only one (case 7-1) had more than 50% of the orthodontists recommend Invisalign® as an option. This patient had moderate crowding in the upper arch and less growth potential, while case 7-2 had minimal crowding and more growth potential. Functional appliances were suggested for both of these patients, but more for case 7-2. The patient with more need for functional appliances seemed to have a lower rate of recommendations for Invisalign®. In the adult Class II division 2 category, the patient with less need for extractions had a higher rate of recommendations for Invisalign®.

Finally, in the Class III patients, it was shown that the patients with less severe A-P discrepancies had higher rates of recommendations for Invisalign®. The cases requiring surgery were not judged to be good candidates for Invisalign®.

The results shown in Table 6 outline the reasons given for not recommending Invisalign® in the total treatment plans. The highest one was extraction plan (36%), and the second one was surgery (23%). Others were concerned about the severity of case, anchorage, and A-P correction.

**Table 6:** The percentages of the reasons given for not recommending Invisalign®

Reasons to not recommend Invisalign®	Percentage
Deepbite, deep curve of Spee	3
A-P correction	8
Surgery case	23
Extraction case	36
7’s not erupted	1
Rotation correction	2
Difficult case	12
Crossbite	3
Anchorage, control of movement	10
Open bite	2
Total	100

Among those cases which were fairly equal in the recommendation for or against using aligners (cases 1-2, 6-2, 7-1, 8-1, 8-2, 9-2), there was a significant difference among extractions and surgery choices (Table 7). Cases with extractions had significantly fewer Invisalign® recommendations (15%) compared to cases with no extractions (55%) (*p* = 0.0015). Additionally, cases with surgery had significantly fewer Invisalign® recommendations (29%) compared to cases with no surgery (57%) (*p* = 0.035).

**Table 7:** Extraction, surgery, and Invisalign® choices

	Invisalign®			*p* value*
	No Inv	Inv	% Inv	
Extractions				
No	37	46	55	0.0015
Yes	11	2	15	
Surgery				
No	31	41	57	0.035
Yes	17	7	29	

*Logistic regression model with GEE

## Discussion

This study was performed to assess what types of malocclusions orthodontists felt would be suitable for treatment with aligners, and also to determine if using digital versus plaster models would affect treatment plans. It has been reported that digital models are an adequate replacement for plaster models with respect to treatment planning for fixed appliance therapy [13, 14], but no one has assessed this for aligner therapy. In the present study, we did not find a difference in decisions regarding the use of aligners with plaster versus digital models. Good intra-rater agreement was observed when aligners were chosen as a treatment option with all combinations of digital or plaster models.

Table 5 shows that the most common reasons for not recommending Invisalign® were extractions (36%), orthognathic surgery (23%), difficult tooth movements (12%), concerns about anchorage (10%), and A-P correction (10%). This supports the finding of Phan and Ling’s publication in 2007 that stated the following conditions were contra-indicated for aligners: crowding and spacing over 5 mm, skeletal anterior-posterior discrepancies of more than 2 mm, centric-relation and centric-occlusion discrepancies, open bites, severely rotated teeth, extrusion of teeth, severely tipped teeth, teeth with short clinical crowns, and arches with multiple missing teeth [1]. Table 7 also reveals that cases with extractions or surgery had fewer Invisalign® recommendations compared to cases with no extractions or surgery.

When treating crowded cases with extractions, it is critical to be aware of canine position, mesio-distal root tipping adjacent to the extraction sites during space closure, incisor torque, and rotation of teeth. In the present study, it was shown that cases requiring extractions were not judged to be good candidates for Invisalign®. In 2003, Bollen, et al. reported that only 29% of those with two or more premolars extracted were able to complete space closure with the initial series of aligners, and none completed the overall treatment [15]. In a prospective study, Kravitz, et al. reported maxillary and mandibular canines achieved only about one third of the predicted rotation in a prospective study [2]. However, in 2015, Li, et al. reported that Invisalign® appliances were as successful as fixed appliances in correcting Class I adult extraction cases by assessing the overall improvement in the ABO-OGS scores in a multicenter randomized controlled trial. OGS scores were similar for alignment, occlusal relations, interproximal contacts, and root angulation, but Invisalign® OGS scores were not as good as braces in reference to occlusal contacts and buccolingual inclination [4].

When treating Class II and Class III cases, surgery or camouflage decisions (with or without extractions) were based on the severity of the A-P discrepancy. In this study, it was shown that practitioners were reluctant to treat cases requiring surgery with Invisalign®. In surgical cases, fixed appliances are helpful for efficient dental decompensation and inter-arch fixation during surgery. Although there are case reports of treatment with aligners for orthognathic surgery cases [16], many orthodontists feel that fixed appliances are more effective to control post-surgical tooth movement and inter-arch coordination.

Besides extraction and surgery, this study found that orthodontists were also concerned about anteroposterior correction. It has been reported that molar distalization in mild Class II malocclusions can be achieved with clear aligners [3, 5, 17]. Some authors have reported low success rates with Invisalign® for correcting large A-P discrepancies. In the official clinical guide of Invisalign® treatment [18], it is recommended that for Class II cases, the amount of A-P correction be limited to 2–4 mm. This correction is largely from dental, not skeletal, changes. For severe Class II malocclusions (4 mm+), Invisalign® recommends using sagittal correctors prior to starting aligner therapy. In the official clinical guide of Invisalign® treatment for Class III cases [19], the amount of Class III correction that can be achieved with aligners is under 2 mm. This amount of anteroposterior correction can be achieved in several ways, including precision cuts for elastics, sequential staging of tooth movements, optimized attachments, and incorporation of TADs. Likewise, large tooth movements may be challenging, as each aligner typically has less than 0.25 mm of programmed tooth movement [2, 3, 5]. Teeth that need significant rotation or translation will require many aligners to accomplish these movements. Vertical and transverse problems may also be difficult to correct, as indicated by the low number of recommendations for aligners when patients displayed these characteristics.

According to a clinical study in 2009, the mean accuracy of tooth movement with Invisalign® was 41% [2]. During the past few years, Align Technology has launched new materials, techniques, and software to expand the scope of tooth movement, the management of extractions, the ability to correct anteroposterior relationships, and the correction of vertical problems [20–22]. In 2011, Align Technology claimed that Invisalign® G4 attachments and SmartForce features provided greater root tip control for canines and central incisors, mesio-distal root uprighting, space closure, and improved optimized extrusion attachments for anterior open bites [20]. In 2013, Invisalign® G5 featured more predictable deepbite correction by using new SmartForce attachments, precision bite ramps, pressure areas for the incisors and canines, and optimized premolar attachments for retention and extrusion to level the curve of Spee [21]. In 2014, Invisalign® G6 was engineered to improve clinical outcomes for orthodontic treatment of severe crowding and bimaxillary protrusion which require premolar extraction and maximum anchorage [22]. The new SmartStage technology, SmartForce, optimized retraction attachments, and optimized anchorage attachments are designed to eliminate unwanted tipping and anterior extrusion during retraction, to improve bodily movement during canine retraction, and to maximize posterior anchorage [22].

In this study, approximately 70% of the orthodontists had been in practice for more than 20 years, and half of the orthodontists were routinely treating patients with aligners. Only 3 cases out of 20 cases (15%) had more than half of the practitioners recommend clear aligners. These cases tended to be the less severe malocclusions. While this percentage may seem low, including more challenging malocclusions allowed us to better investigate the reasons that aligners were not recommended.

Previous publications on the Invisalign® technique have mainly covered technical aspects or materials or have presented results from case reports or case series. Orthodontists often recommend aligner therapy to patients based on their past experiences. In this study, most orthodontists avoided recommending aligners to patients requiring extractions, surgery, or difficult tooth movements. While Align Technology continues to release new materials, software, and features to broaden the scope of Invisalign® care, orthodontists may still be basing their recommendations from their experience with earlier Invisalign® materials and techniques. Thus, Invisalign® may need to provide orthodontists with additional education and evidence in order to change their opinions regarding case selection.

## Conclusions

In this study, viewing orthodontic records with digital versus plaster models did not influence decisions about Invisalign® recommendations. Additionally, our sample of orthodontists tended to not recommend Invisalign® to treat extraction cases, surgical cases, or difficult cases.

## Abbreviations

GEE: Generalized estimating equations regression
